# A possible macronova in the late afterglow of the long–short burst GRB 060614

**DOI:** 10.1038/ncomms8323

**Published:** 2015-06-11

**Authors:** Bin Yang, Zhi-Ping Jin, Xiang Li, Stefano Covino, Xian-Zhong Zheng, Kenta Hotokezaka, Yi-Zhong Fan, Tsvi Piran, Da-Ming Wei

**Affiliations:** 1 Key Laboratory of Dark Matter and Space Astronomy, Purple Mountain Observatory, Chinese Academy of Sciences, Nanjing 210008, China; 2 University of Chinese Academy of Sciences, Yuquan Road 19, Beijing 100049, China; 3 INAF/Brera Astronomical Observatory, via Bianchi 46, I-23807 Merate, Italy; 4 Racah Institute of Physics, The Hebrew University, Jerusalem 91904, Israel; 5 Collaborative Innovation Center of Modern Astronomy and Space Exploration, Nanjing University, Nanjing 210046, China

## Abstract

Long-duration (>2 s) γ-ray bursts that are believed to originate from the death of massive stars are expected to be accompanied by supernovae. GRB 060614, that lasted 102 s, lacks a supernova-like emission down to very stringent limits and its physical origin is still debated. Here we report the discovery of near-infrared bump that is significantly above the regular decaying afterglow. This red bump is inconsistent with even the weakest known supernova. However, it can arise from a Li-Paczyński macronova—the radioactive decay of debris following a compact binary merger. If this interpretation is correct, GRB 060614 arose from a compact binary merger rather than from the death of a massive star and it was a site of a significant production of heavy r-process elements. The significant ejected mass favours a black hole–neutron star merger but a double neutron star merger cannot be ruled out.

## Abstract (web summary)

The gamma-ray burst GRB 060614 was an unusual astrophysical event whose origins are still unclear. This study re-examines the burst's afterglow data and finds an excess in the spectrum that appears to be consistent with a weak macronova, suggesting that GRB 060614 originated from a compact binary merger.

## Graphical abstract

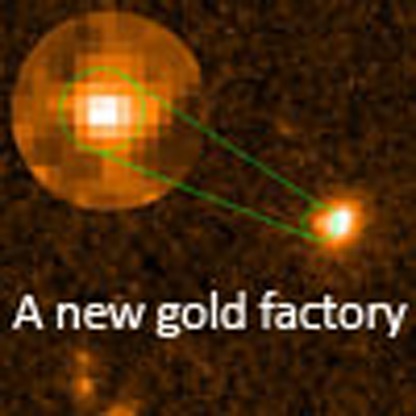

Long-duration (>2 s) γ-ray bursts (GRBs) are believed to originate from Collapsars that involve death of massive stars and are expected to be accompanied by luminous supernovae (SNe). GRB 060614 was a nearby burst with a duration of 102 s at a redshift of 0.125(ref. 1). While it is classified as a long burst according to its duration, extensive searches did not find any SNe-like emission down to limits hundreds of times fainter^2^,^3^,^4^ than SN 1998bw, the archetypal hypernova that accompanied long GRBs^5^. Moreover, the temporal lag and peak luminosity of GRB 060614 fell entirely within the short duration subclass and the properties of the host galaxy distinguish it from other long-duration GRB hosts. Thus, GRB 060614 did not fit into the standard picture in which long-duration GRBs arise from the collapse of massive stars while short ones arise from compact binary mergers. It was nicknamed the ‘long–short burst' as its origin was unclear. Some speculated that it originated from compact binary merger and thus it is intrinsically a ‘short' GRB^1^,^4^,^6^,^7^,^8^. Others proposed that it was formed in a new type of a Collapsar which produces an energetic γ-ray burst that is not accompanied by an SNe^2^,^3^,^4^.

Two recent developments may shed a new light on the origin of this object. The first is the detection of a few very weak SNe (for example, SN 2008ha^9^) with peak bolometric luminosities as low as *L*∼10^41^ erg s^−1^. The second is the detection of an infrared bump, again with a *L*∼10^41^ erg s^−1^, in the late afterglow of the short burst GRB 130603B^10^,^11^. This was interpreted as a Li-Paczyński macronova (also called kilonova)^12^,^13^,^14^,^15^,^16^,^17^,^18^,^19^—a near-infrared/optical transient powered by the radioactive decay of heavy elements synthesized in the ejecta of a compact binary merger. Motivated by these discoveries, we re-examined the afterglow data of this peculiar burst searching for a signal characteristic to one of these events.

The X-ray and UV/optical afterglow data of GRB 060614, were extensively examined in the literature^20^,^21^ and found to follow very well the fireball afterglow model up to *t*∼20 days^22^. The *J*-band has been disregarded because only upper limits ∼19–20^th^ mag with a sizeable scatter are available at *t* >2.7 days, and these are too bright to significantly constrain even supernovae as luminous as SN 1998bw^23^. In this work we focus on the optical emission. We have re-analysed all the late time (that is, *t* ⩾1.7 days) very large telescope (VLT) *V*, *R* and *I* -band archival data and the Hubble space telescope (HST) F606W and F814W archival data, including those reported in the literature^3^,^4^ and several unpublished data points. Details on data reduction are given in the Methods.

## Results

### The discovery of a significant F814W-band excess

**Figure 1:**
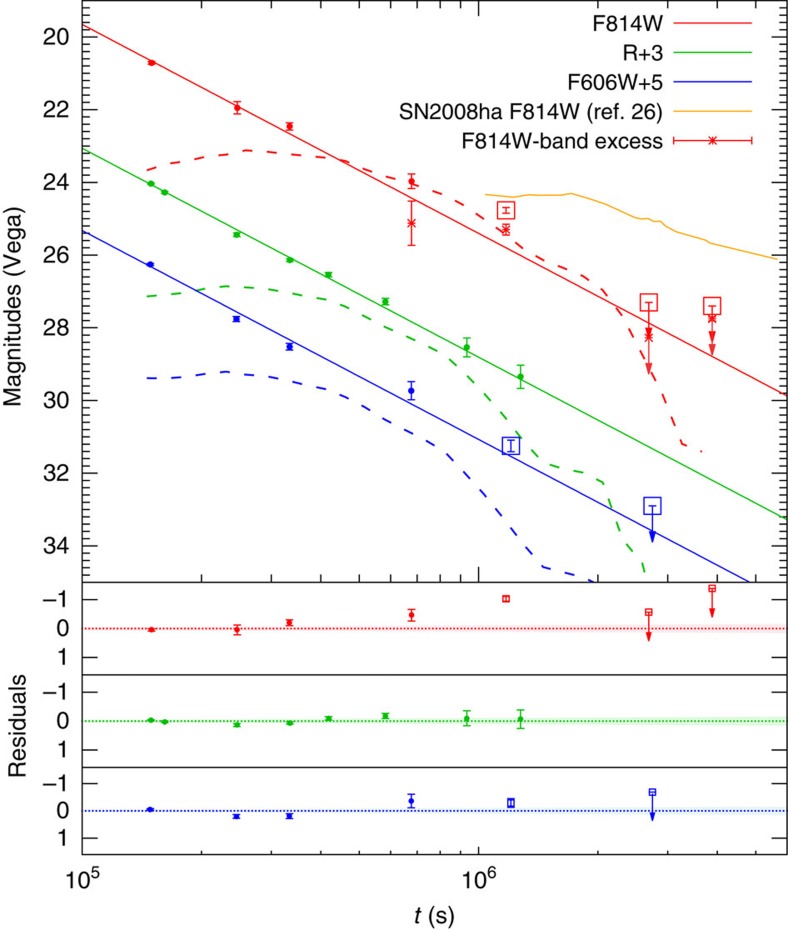
The afterglow emission of GRB 060614. The VLT and HST observation vega magnitudes including their 1*σ* statistical errors of the photon noise and the sky variance and the 3*σ* upper limits (the downward arrows) are adopted from Supplementary Table 1. The small amounts of foreground and host extinction have not been corrected. Note that the VLT *V*/*I* band data have been calibrated to the HST F606W/F814W filters with proper *k*-corrections (see Methods). The VLT data (the circles) are canonical fireball afterglow emission while the HST F814W detection (marked in the square) at *t*∼13.6 days is significantly in excess of the same extrapolated power-law decline (see the residual), which is at odds with the afterglow model. The F814W-band light curve of SN 2008ha 27 expected at *z*=0.125 is also presented for comparison. The dashed lines are macronova model light curves generated from numerical simulation 28 for the ejecta from a black hole–neutron star merger. Error bars represent s.e.

Figure 1 depicts the most complete late-time optical light curves (see Supplementary Table 1; the late VLT upper limits are not shown in Fig. 1) of this burst. The VLT *V*, *R* and *I*-band fluxes decrease with time as ∝*t*^−2.30±0.03^ (see Fig. 1, in which the VLT *V*/*I* band data have been calibrated to the F606W/F814W filters of HST with proper *k*-corrections), consistent with that found earlier^3^,^20^,^21^. However, the first HST F814W data point is significantly above the same extrapolated power-law decline. The significance of the deviation is ∼6*σ* (see the estimate in the Methods). No statistically significant excess is present in both the F606W and the *R* bands. The F814W-band excess is made most forcibly by considering the colour evolution of the transient, defined as the difference between the magnitudes in each filter, which evolves from *V–I*≈0.65 mag by the VLT (correspondingly for HST we have F606W–F814W≈0.55 mag) at about *t* ∼1.7 days to F606W–F814W≈1.5 mag by HST at about 13.6 days after the trigger of the burst. With proper/minor extinction corrections, the optical to X-ray spectrum energy distribution for GRB 060614 at the epoch of ∼1.9 days is nicely fitted by a single power law^3^,^20^,^21^ *F*_v_∝*v*^−0.8^. In the standard external forward shock afterglow model, the cooling frequency is expected to drop with time as^22^ *v*_c_∝*t*^−1/2^. Thus, it cannot change the optical spectrum in the time interval of 1.9–13.6 days. Hence, the remarkable colour change and the F814W-band excess of ∼1 mag suggest a new component. Like in GRB 130603B this component was observed at one epoch only. After the subtraction of the power-law decay component, the flux of the excess component decreased with time faster than *t*^−3.2^ for *t* >13.6 days. Note that an unexpected optical re-brightening was also detected in GRB080503, another ‘long–short' burst^24^. However, unlike the excess component identified here, that re-brightening was achromatic in optical to X-ray bands and therefore likely originated by a different process.

## Discussion

**Figure 2:**
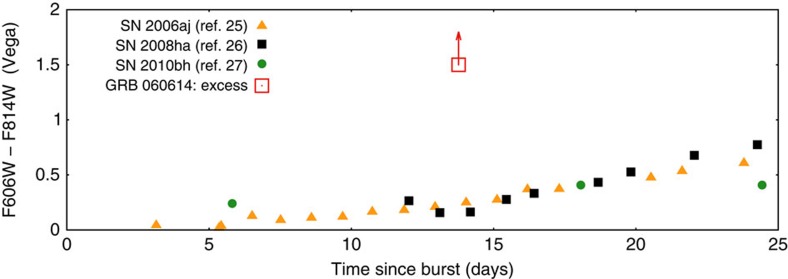
The colour change of some supernovae in comparison with our excess component. The emission of SN 2006aj, SN 2008ha and SN 2010bh, adopted from the literature^25^,^26^,^27^ has been shifted to *z*=0.125, the redshift of GRB 060614, with corrections on the time, frequency and extinction. Note that the ‘excess component' is much redder than them (the upward arrow represents a lower limit).

Shortly after the discovery of GRB 060614 it was speculated that it is powered by an ‘unusual' core collapse of a massive star^2^,^3^. We turn now to explore whether the F814W-band excess can be powered by a weak supernova. Figure 2 depicts the colour F606W–F814W of the excess component (we take F606W–F814W≈1.5 mag as a conservative lower limit of the colour of the ‘excess' component due to the lack of simultaneous excess in F606W-band) with that of SN 2006aj^25^, SN 2008ha (i.e., the extremely dim event)^26^ and SN 2010bh^27^. The excess component has a much redder spectrum than the three supernovae. If the ‘excess component' was thermal it had a low effective temperature *T*_eff_ <3,000 K to yield the very soft spectrum. Such unusually low effective temperature is also needed to account for the very rapid decline of the excess component. The expansion velocity can be estimated as *υ*∼1.2 × 10^4^ km s^−1^ (*L*/10^41^ erg s^−1^)^1/2^(*T*_eff_/3,000 K)^−2^(*t*/13.6 days)^−1^. The implied ^56^Ni mass is 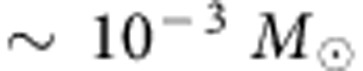 if this was a supernova-like event that peaked at ∼13.6 days^9^. We take a standard cosmology model with *H*_0_=71 km s^−1^ Mpc^−1^, *Ω*_M_=0.32 and *Ω*_Λ_=0.68. Comparing with the extremely faint SN 2008ha after proper corrections to *z*=0.125, the peak F814W-band emission of the ‘excess component' is lower by ∼1 mag and the decline is also much faster. Hence the ‘excess component' is remarkably different from SN 2008ha.

The low luminosity as well as the low effective temperature of the transient emission are typical characteristics of a macronova, a transient arising from the radioactive *β*-decay of material ejected in a compact binary merger. The opacity of the macronova material is determined by the Lanthanides that are produced via r-process in the neutron-rich outflow. This opacity is very large (*κ*≈10 cm^2^ g^−1^) resulting in a weak, late and red emission. The emerging flux is greatly diminished by line blanketing, with the radiation peaking in the near-infrared and being produced over a timescale of ∼1–2 weeks^17^,^18^. Simple analytic estimates, using a radioactive β-decay heating rate^16^,^28^ of 10^10^ erg s^−1^ g^−1^[*t*/(1+*z*)1 day]^−1.3^, suggest that in order to explain the observed F814W-band excess, the required ejecta mass and expansion velocity are: 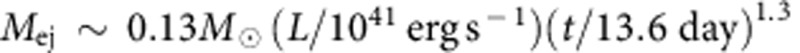 and *υ*∼0.1*c* (*L*/10^41^ erg s^−1^)^1/2^(*T*_eff_/2,000 K)^−2^(*t*/13.6 days)^−1^, respectively. Note that the macronova outflow is quite cold at such a late time^17^,^18^. The effective temperature is *T*_eff_≈2,000 K and the observer's F814W-band is above the peak of the black body spectrum. The emitting radius and the corresponding expansion velocity are much larger than in a supernova at this stage. Scaled up numerical simulations of lighter ejecta from black hole–neutron star mergers^28^ suggest that 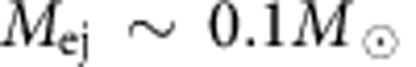 and a velocity ∼02*c* can account for the observed F814W-band excess. This numerical example is presented in Fig. 1 in dashed lines.

The implied ejecta mass is large compared with the mass ejection estimated numerically to take place in double neutron star mergers. However, it is within the possible range of dynamical ejecta of black hole–neutron star mergers with some extreme parameters (a large neutron star radius and a high black hole spin aligned with the orbital angular momentum)^14^,^29^,^30^,^31^,^32^. An accretion disk wind may contribute some additional mass as well^15^,^33^,^34^. However, the radioactive heating due to fission of the heavy r-process nuclei, which is quite uncertain and subdominant in current heating estimates^16^, may play an important role in the energy deposition. It may increase the energy deposition rate at around 10 days by a significant factor^35^. This may reduce the required ejecta mass to 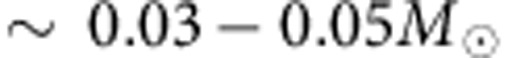. This range of the ejecta masses is well within the range of the dynamical ejecta of black hole–neutron star mergers and it is even compatible with some estimates of double neutron star mergers.

We conclude that while a weak supernova cannot explain the observations, a high mass ejection macronova may. Like in GRB 130603B we must caution here that this interpretation is based on a single data point. However, if this interpretation is correct, it has far reaching implications. First, the presence of macronovae in both the canonical short burst GRB 130603B and in this ‘long–short' one, GRB 060614, suggests that the phenomenon is common and the prospects of detecting these transients are promising. A more conclusive detection based on more than a single data point could be achieved in the future provided that denser HST observations are carried out. Moreover, as a black hole–neutron star merger is favoured in explaining the large ejected mass this implies that such binary systems may exist and their mergers are also responsible for GRBs. It also suggests that the ‘long–short' burst was in fact ‘short' in nature, namely, it arose from a merger and not from a Collapsar. The fact that a merger generates a 100 s long burst is interesting and puzzling by itself.

Clearly such events would contribute a significant fraction of the r-process material^36^. The actual contribution relative to the contribution of 130603B-like events is difficult to estimate as it is unclear which fraction of the macronovae/kilonovae behave as each type. Because of beaming most mergers will not be observed as GRBs. However, they emit omnidirectional gravitational radiation that can be detected by the upcoming Advanced LIGO/VIRGO/KAGRA detectors. These near-infrared/optical macronovae could serve as promising electromagnetic counterparts of gravitational wave triggers in the upcoming Advanced LIGO/VIRGO/KAGRA era.

## Methods

### Data reduction

We retrieved the public VLT imaging data of GRB 060614 from European Southern Observatory (ESO) Science Archive Facility (http://archive.eso.org). The raw data were reduced following standard procedures, including bias subtraction, flat fielding, bad pixel removal and combination. Observations made with the same instrument and filter at different epochs are compared with that of the last epoch. The software package ISIS (http://www2.iap.fr/users/alard/package.html) is used to subtract images and measure the GRB afterglow from the residual images. Photometric errors are estimated from the photon noise and the sky variance to 1*σ* confidence level. The 3*σ* of the background root mean square of the residual images is taken as the limiting magnitude. Finally, standard stars observed on 16 June 2006 were used for the absolute calibration. The results are presented in Supplementary Table 1, being well consistent with these given by other groups^3^,^21^. We assumed that the afterglow is characterized by the same power-law spectrum with index *β*=0.80 during these observations^20^, with which we get the *k*-corrections between the VLT *V*/*I* and HST F606W/F814W magnitudes, namely 0.12 mag and 0.02 mag, respectively. Such corrections have been taken into account in Fig. 1.

HST archive data of GRB 060614 are available from the Mikulski Archive for Space Telescopes (MAST; http://archive.stsci.edu), including one observation with the Wide Field and Planetary Camera 2 (WFPC2) and four observations with the Advanced Camera for Surveys (ACS) in F606W and F814W bands. The reduced data provided by MAST were used in our analysis. The last observation has been taken as the reference and the other images of the same filter are subtracted in order to directly measure fluxes of the afterglow from the residual images. Empirical point spread functions (PSFs) were built with bright stars in each image. Bright compact objects in the same field were used to align and relatively calibrate these images. WFPC2 image differs from ACS image in PSF. Before image subtraction, the WFPC2 and ACS images were matched to the same resolution by convolving each with the other's PSF. The PSF-matched WFPC2 and ACS images were aligned and subtracted. Aperture photometry was carried out for the afterglow in the residual image. The aperture correction derived from the empirical PSF was applied to yield the total flux. The host galaxy was used to relatively calibrate the afterglow between images, and the ACS zeropoints were used for absolute calibration. If the signal of the afterglow is too faint to be a secure detection, an upper limit of 3*σ* background root mean square is adopted. The results are reported in Supplementary Table 1, being well in agreement with these published in the literature^4^. The magnitudes of the host galaxy are measured in the last observation of all filters and can well be fitted by an Sc type galaxy template (Supplementary Fig. 1), demonstrating the self-consistence of our results.

### VLT light curve decline rate and significance of the excess

As found in previous studies, the late-time optical/X-ray afterglow emission of GRB 060614 can be interpreted within the fireball forward shock model^20^,^21^. Motivated by such a fact, we assume that the *I*, *R* and *V* light curves follow the same power-law decline. In our fit there are four free parameters, three are related to the initial flux/magnitude in these three bands and the last is the decline rate needed in further analysis. We fitted all the VLT data (combined *I*, *R* and *V* band together) during the first 15 days (after which there are just upper limits) to determine these four parameters as well as their errors. The best-fit decline is found to be ∝*t*^−2.3±0.03^, well consistent with that obtained in optical to X-ray bands in previous studies^3^,^20^,^21^. As a result of the propagation of uncertainties, the errors of the best-fit light curves are consequently inferred (the shadow regions in the residual plot of Fig. 2 represent the 1*σ* errors of the best-fit light curves). Please note that in Fig. 2 the VLT *V*/*I* band emission have been calibrated to HST F606W/F814W filters with proper *k*-corrections. The flux separation between the HST F814W-band data and the fitted curve at *t*∼13.6 days is *F*_excess_=0.182 *μ*Jy. The flux error of the F814W-band emission at *t*∼13.6 days is *δF*_obs_≈0.024 μJy. The flux error of the best fitted F814W-band light curve at *t*∼13.6 days is *δF*_fit_≈0.012 μJy. The significance of the excess component is estimated by 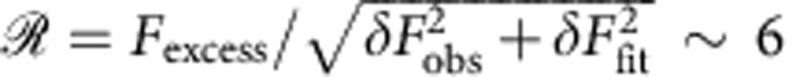. We therefore suggest that the excess component identified in this work is statistically significant at a confidence level of ∼6*σ*.

## Additional information

**How to cite this article:** Yang, B. *et al*. A possible macronova in the late afterglow of the long–short burst GRB 060614. *Nat. Commun.* 6:7323 doi: 10.1038/ncomms8323 (2015).

## Supplementary Material

Supplementary Information Supplementary Figure 1, Supplementary Table 1 and Supplementary References
