# Supplementary material for: A possible macronova in the late afterglow of the long–short burst GRB 060614
Source: Nat Commun. 2015 Jun 11;6:7323. doi: 10.1038/ncomms8323 (PMC4490351; doi:10.1038/ncomms8323)
Supplement: Supplementary Information — Supplementary Figure 1, Supplementary Table 1 and Supplementary References [file ncomms8323-s1.pdf]

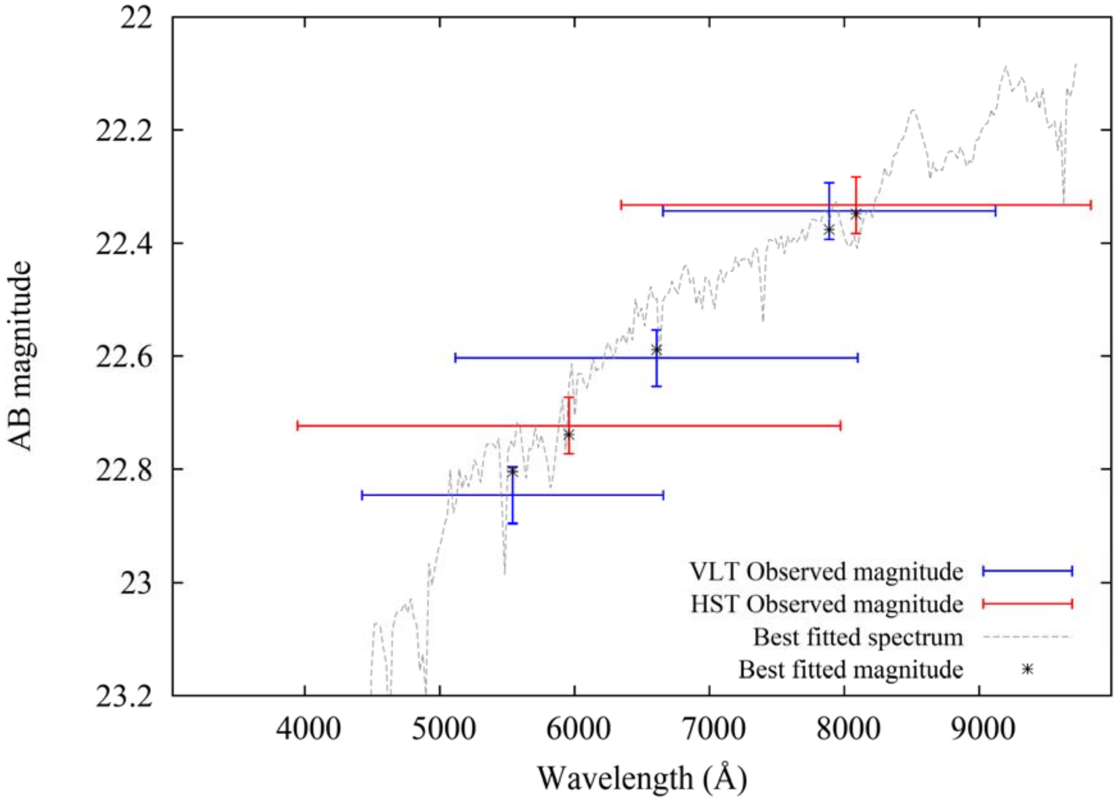

Supplementary Figure 1. **The observations of the host galaxy of GRB 060614 by VLT and HST.** Following [1] we fit the host galaxy magnitudes by an Sc type template. The redshift of the host galaxy z=0.125 and the Galactic extinction A_V=0.07 mag have been taken into account.

Supplementary Table 1. Log of observations

Time from GRB[a] (days)	Filter	Exposure time (s)	Instrument	Magnitude[b]
1.72034	V	2×120	VLT+FORS1	21.38±0.03
2.83515	V	4×90	VLT+FORS1	22.88±0.07
3.86077	V	2×120+4×180	VLT+FORS1	23.64±0.09
7.8279	V	3×180	VLT+FORS2	24.85±0.25
23.79964	V	2×120	VLT+FORS1	>24.8
32.78644	V	5×120	VLT+FORS1	[22.96±0.04]
108.57188	V	3×300	VLT+FORS2	[22.87±0.04]
1.72583	R	2×120	VLT+FORS1	21.03±0.02
1.86974	R	2×120	VLT+FORS1	21.28±0.03
2.84199	R	2×120	VLT+FORS1	22.44±0.05
3.86899	R	2×120+4×180	VLT+FORS1	23.14±0.04
4.84365	R	2×180	VLT+FORS1	23.54±0.06
6.74083	R	3×180	VLT+FORS1	24.28±0.09
10.81441	R	2×300	VLT+FORS1	25.54±0.26
14.77259	R	4×300+4×180	VLT+FORS1	26.35±0.32
19.67818	R	6×240	VLT+FORS1	>26.3
23.80494	R	2×120	VLT+FORS1	>24.6
32.79667	R	3×180	VLT+FORS1	>25.4
44.73601	R	5×240+2×180	VLT+FORS1	>26.3
64.70367	R	12×300	VLT+FORS1	[22.42±0.03]
1.73236	I	3×120	VLT+FORS1	20.73±0.04
2.84826	I	3×120	VLT+FORS1	21.97±0.17
3.8584	I	4×300	VLT+FORS1	22.48±0.10
7.84052	I	3×120	VLT+FORS2	23.99±0.20
23.81008	I	2×120	VLT+FORS1	>23.9
32.80572	I	3×180	VLT+FORS1	[21.99±0.04]
108.58482	I	4×240	VLT+FORS2	[21.94±0.04]
13.97023	F606W	6000	HST+WFPC2	26.25±0.16
31.76674	F606W	3600	HST+ACS	>27.9
85.59018	F606W	4372	HST+ACS	[22.66±0.02]
13.57128	F814W	6000	HST+WFPC2	24.77±0.08
31.09855	F814W	3600	HST+ACS	>27.3
44.95641	F814W	4840	HST+ACS	>27.4
139.44208	F814W	4840	HST+ACS	[21.95±0.02]

Note.

a. Time since the burst trigger, which occurred at 2006 July 14, 12:43:48 UT.

b. These values have not been corrected for the Galactic extinction of A_V = 0.07 mag. The magnitudes in square brackets are for the host galaxy. The definitions of the errors and upper limits are described in the Methods (note that 0.02 magnitude of uncertainty in absolutely calibrations has been added to the statistical errors).

Supplementary References

[1] A. Gal-Yam, et al. A novel explosive process is required for the γ-ray burst GRB 060614. *Nature* **444**, 1053-1055 (2006).
